# Supplementary figures and images for: Normal locomotion in zebrafish lacking the sodium channel NaV1.4 suggests that the need for muscle action potentials is not universal
Source: PLoS Biol. 2025 Apr 24;23(4):e3003137. doi: 10.1371/journal.pbio.3003137 (PMC12021243; doi:10.1371/journal.pbio.3003137)

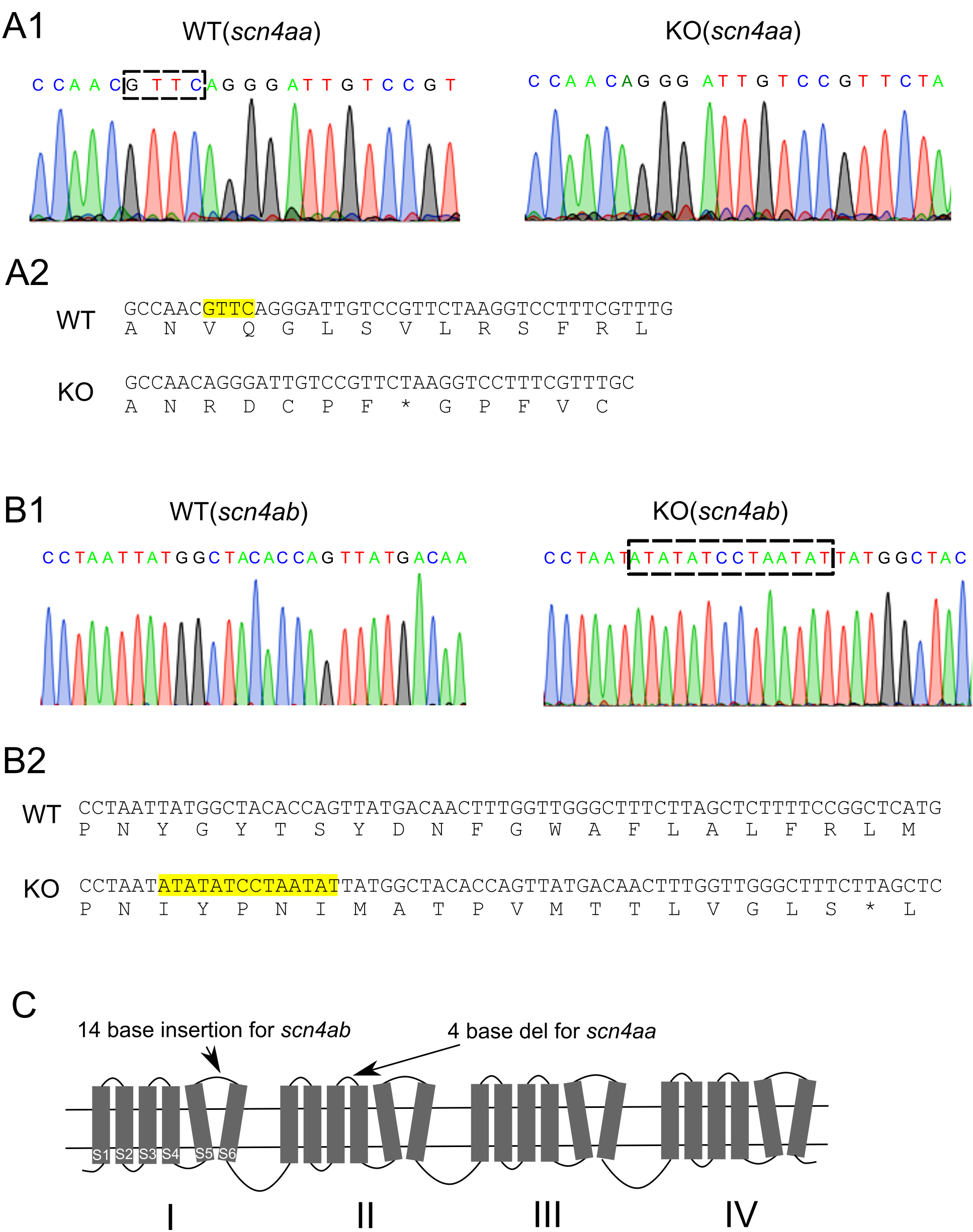

Supplement: S1 Fig — (A1) Genomic sequences of the WT (left) and KO (right) zebrafish around the CRISPR target of scn4aa. The four bases in the dotted box were deleted from the mutant allele. (A2) Encoded amino acid sequences of the WT and KO zebrafish. Deletion of four bases highlighted in yellow engendered a stop codon (asterisk) in KO fish. (B1) Genome sequences of the WT (left) and KO (right) fish around the CRISPR target of scn4ab. The 14 bases in the dotted box were inserted into the mutant allele. (B2) Encoded amino acid sequences of the WT and KO fish. The insertion of the 14 bases highlighted in yellow generated a stop codon (asterisk) in the KO zebrafish. (C) Diagram of NaV with domains I–IV, each containing six transmembrane regions S1–S6. Positions of the mutations are indicated. (TIFF) [file pbio.3003137.s001.tiff]

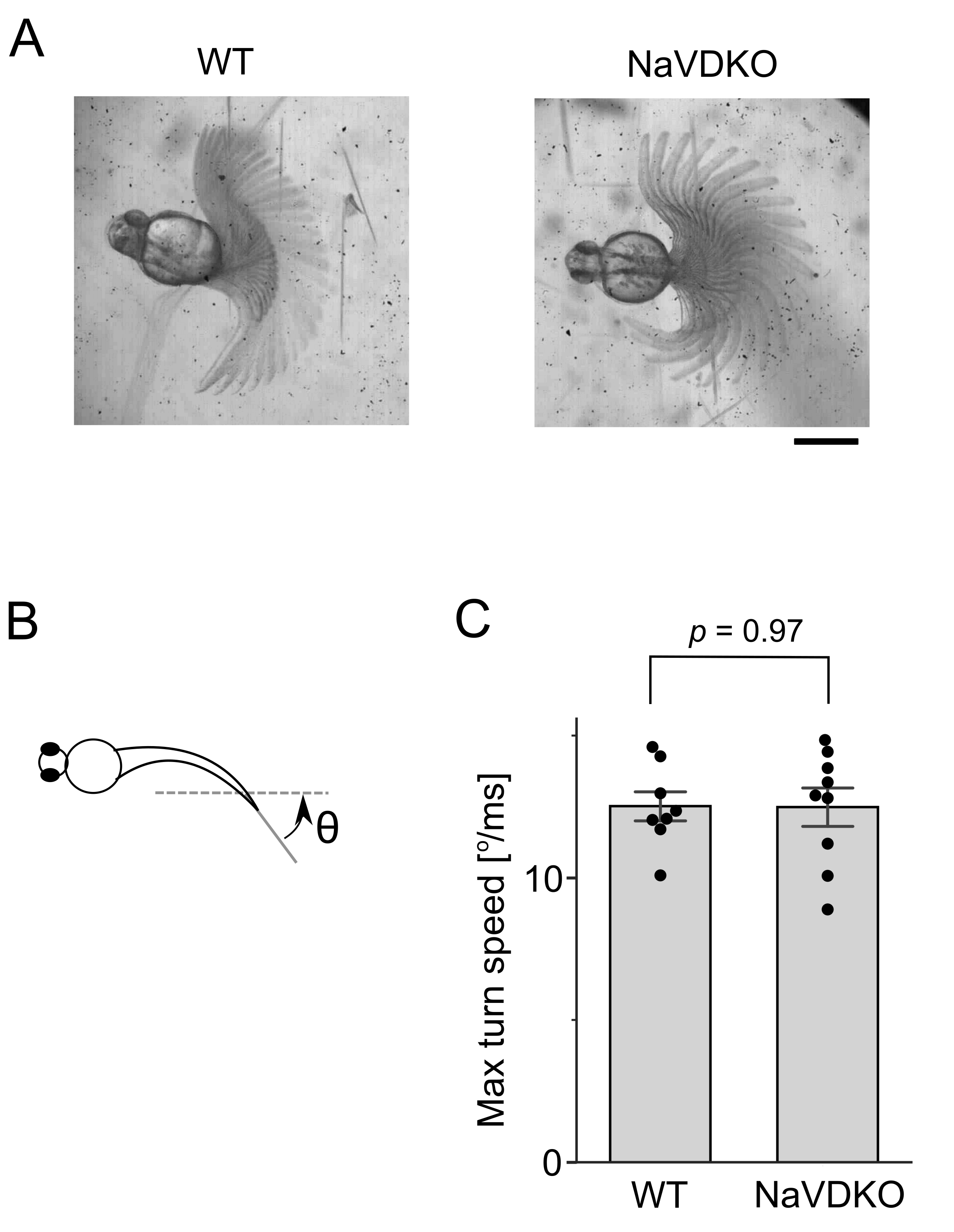

Supplement: S2 Fig — (A) Representative coiling activities of WT and NaVDKO zebrafish. The images were superimposed every 2 ms. Scale bar: 0.5 mm. (B) The angle θ in images was measured as indicated. (C) Plot of maximum turn speed. The numerical data presented in this figure can be found in S1 Data. (TIFF) [file pbio.3003137.s002.tiff]

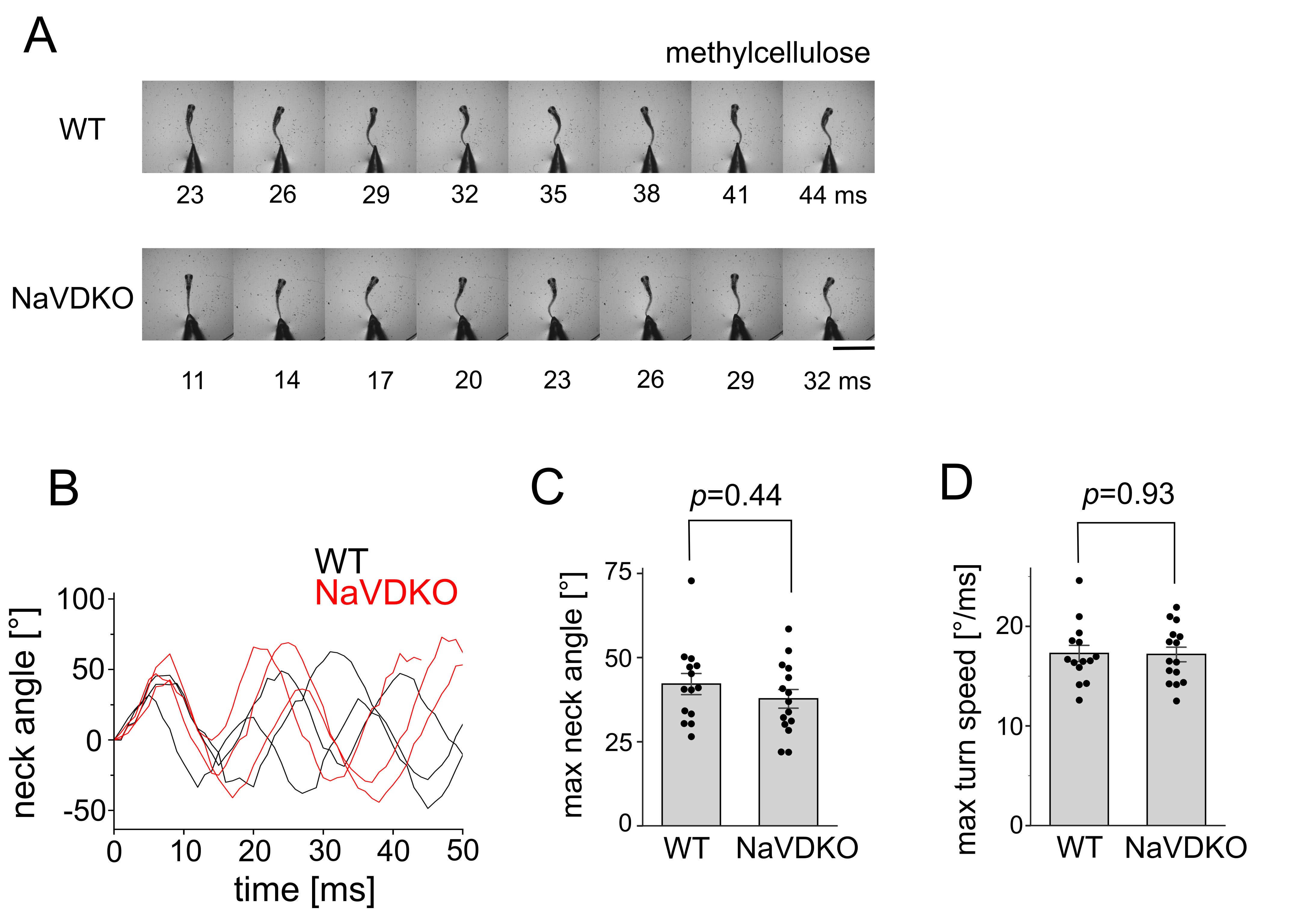

Supplement: S3 Fig — (A) Representative escape responses of WT (upper) and NaVDKO (lower) embryos at 4 dpf in 2% (w/v) methylcellulose. The time elapsed since the start of recording is indicated below the image. Scale bar: 1 mm. (B) Representative plots of the “neck angle” during the escape behavior of WT and NaVDKO fish. (C) Analysis of the maximum neck angle in the first turn of 4 dpf fish (n = 10 for WT, n = 9 for NaVDKO). (D) Analysis of the maximum turn angle speed of 4 dpf fish, calculated from the differential of the neck angle (n = 10 for WT, n = 9 for NaVDKO). The numerical data presented in this figure can be found in S1 Data. (TIFF) [file pbio.3003137.s003.tiff]

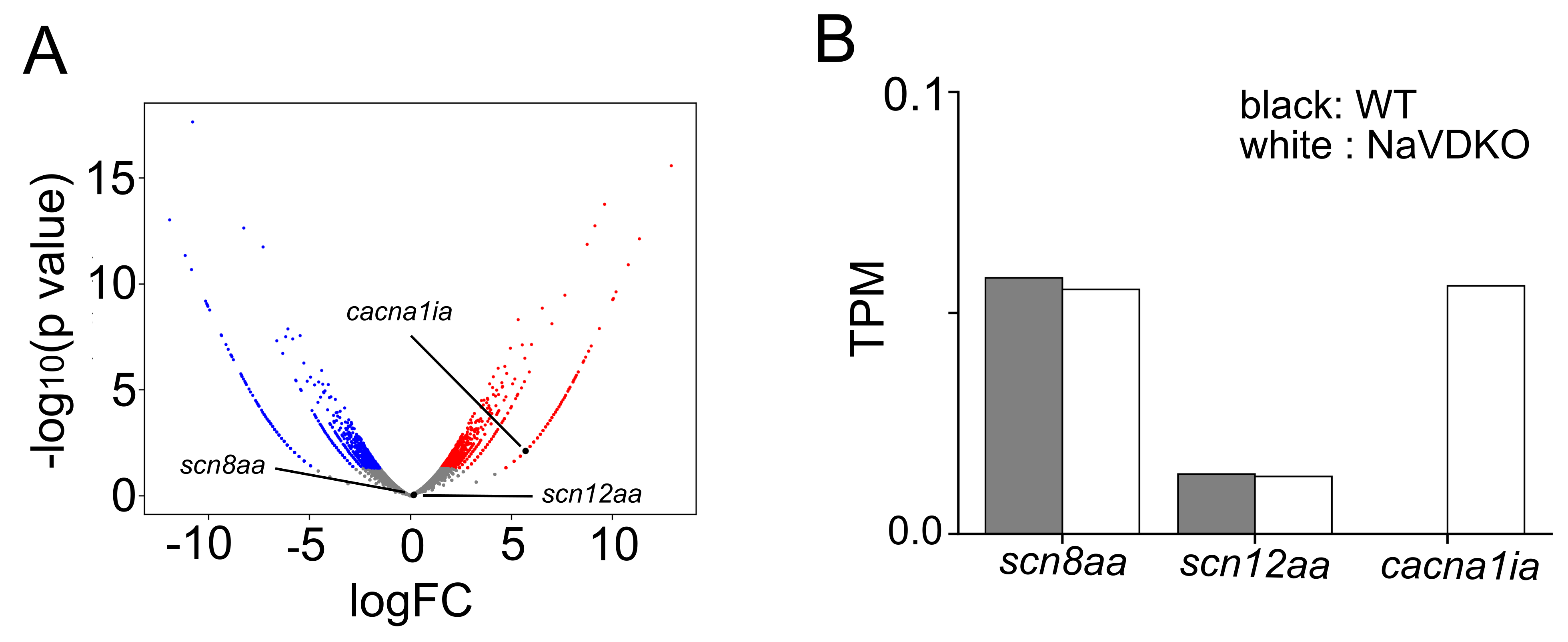

Supplement: S4 Fig — (A) Volcano plot showing the p values of DEGs. Red and blue dots indicate genes with p-values smaller than 0.05. Genes with log(FC) values larger than one and smaller than minus one are shown in red and blue, respectively. The numerical data presented in this figure can be found in S3 Table. (B) Transcripts per million (TPM) values for scn8aa, scn12aa, and cacna1ia. The numerical data presented in this figure can be found in S4 and S6 Tables. (TIFF) [file pbio.3003137.s004.tiff]

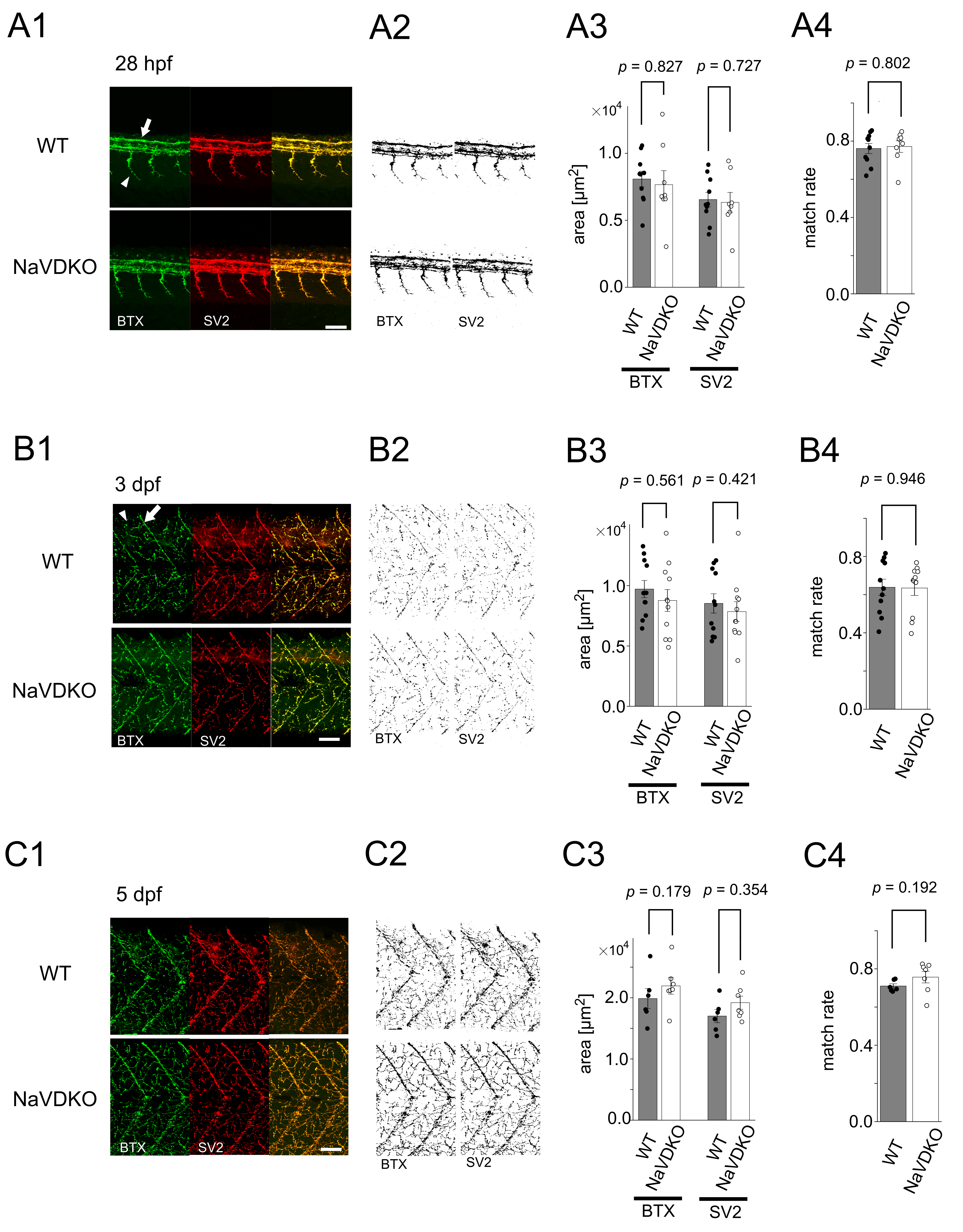

Supplement: S5 Fig — (A1, B1, and C1) BTX and anti-SV2 signals at 28 hpf, 3 dpf, and 5 dpf embryo, respectively. The BTX (left), anti-SV2 (middle), and merged (right) images are shown. The arrow and arrowhead in A1 indicate signals in the spinal cord and middle body segments, respectively. The arrow and arrowhead in B1 indicate signals at the boundaries between body segments and punctate signals in body segments, which are the NMJs of fast muscles. Scale bar: 50 µm. (A2, B2, and C2) Representative images showing pixels above the thresholds obtained from A1, B1, and C1. (A3, B3, and C3) Areas positive for BTX and SV2 in WT and NaVDKO embryos. (A4, B4, and C4) Overlap fractions of the BTX- and SV2-positive pixels. The numerical data presented in this figure can be found in S1 Data. (TIFF) [file pbio.3003137.s005.tiff]

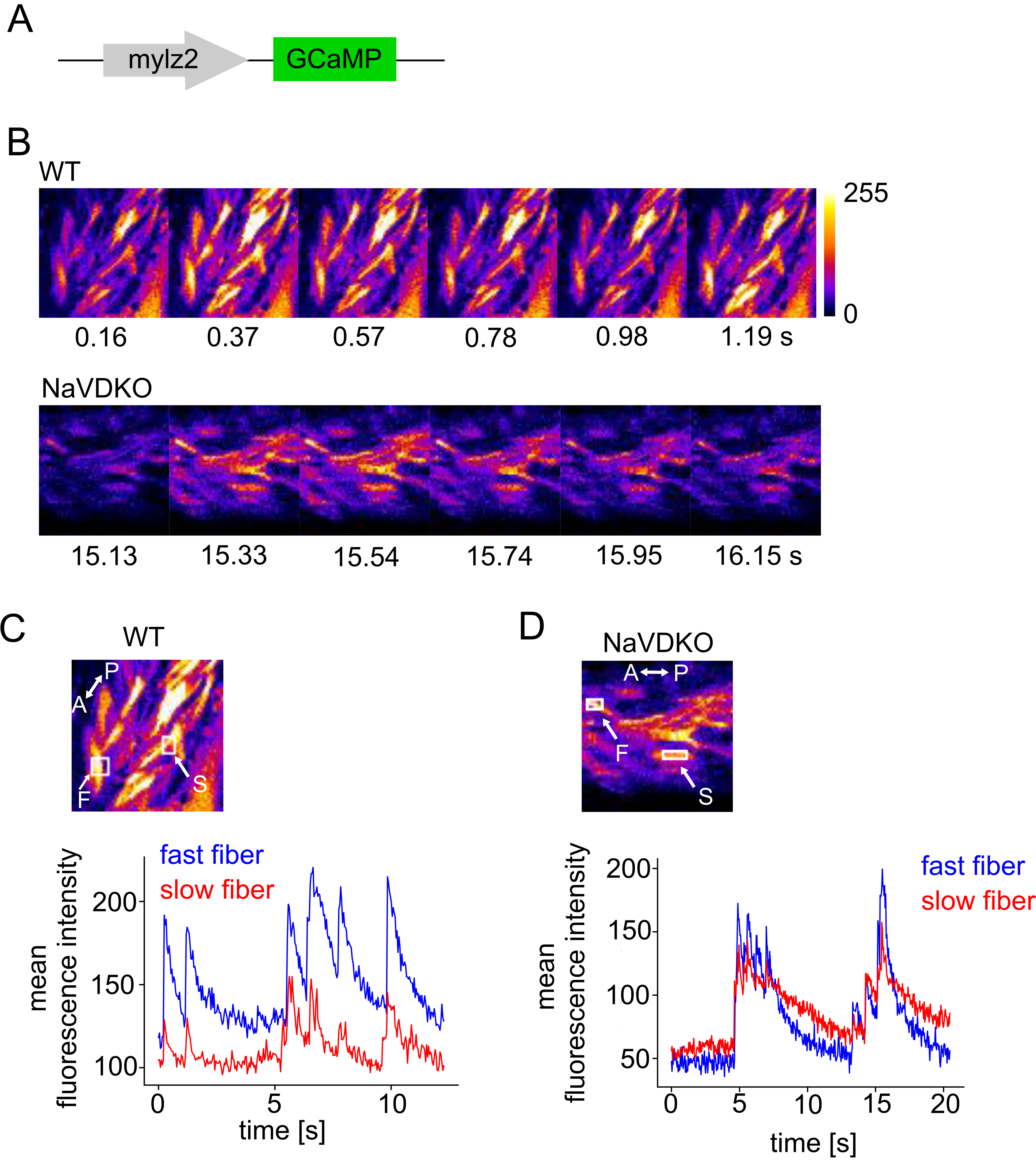

Supplement: S6 Fig — (A) GCaMP7a expression was driven by the mylz2 promoter. (B) Spontaneous muscle excitation in WT (upper) and NaVDKO (lower) fish. The numbers below indicate the time elapsed from the start of the recording. The fluorescence intensity is indicated by color. (C and D) Activities of WT (C) and NaVDKO fish (D). “A” and “P” in the upper panel indicate anterior and posterior, respectively. The mean fluorescence intensity for the pixels in the white boxes is plotted against time in the lower panels. F and S indicate fast and slow fibers, respectively. Fast and slow fibers were identified based on their orientation along the anterior–posterior axis of the body. The numerical data presented in this figure can be found in S1 Data. (TIFF) [file pbio.3003137.s006.tiff]

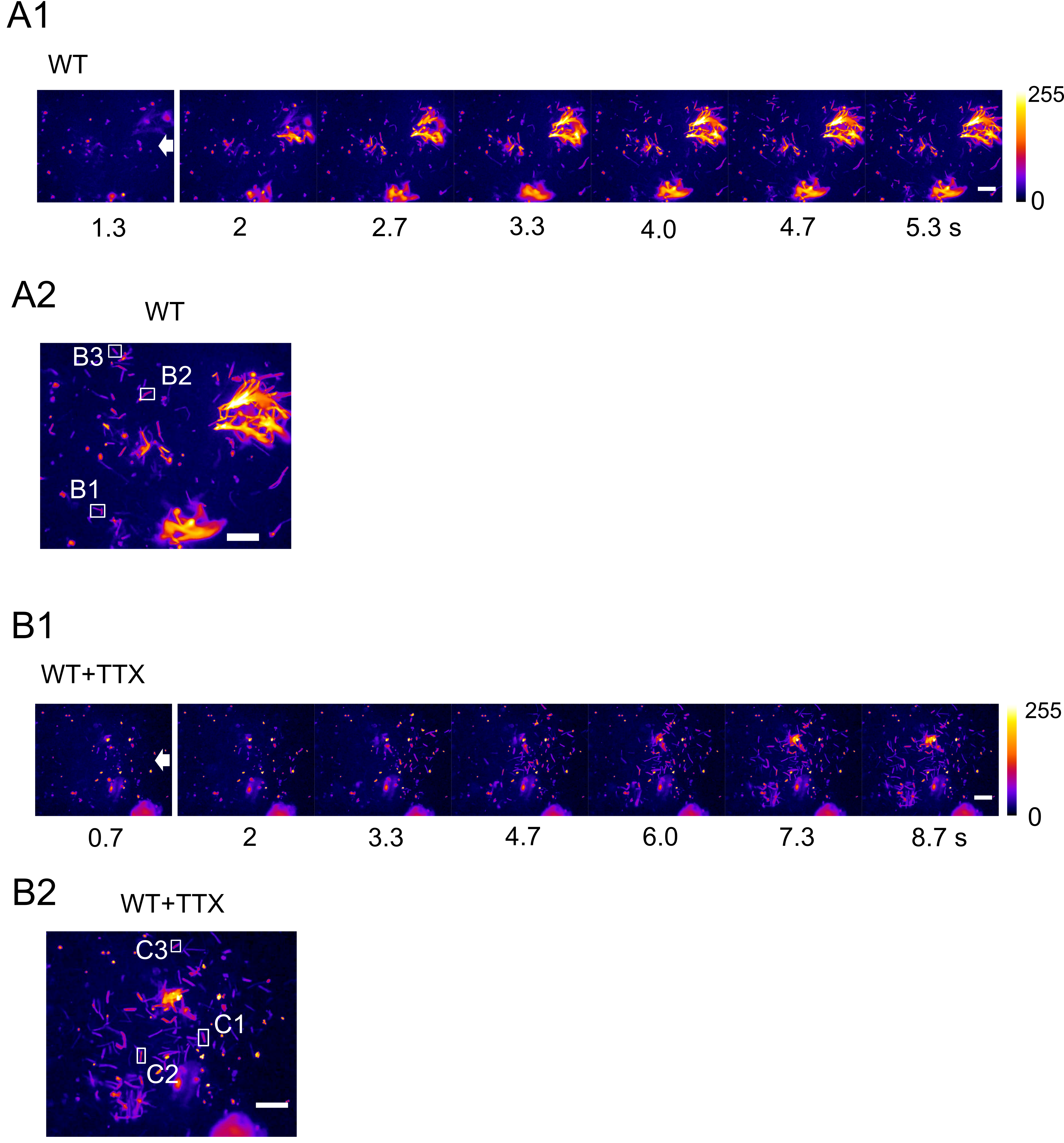

Supplement: S7 Fig — (A1 and B1) Snapshots of field images of the representative Ca2+ imaging of WT in the absence and presence of 1 µ M TTX, respectively. The numbers indicate the time from the beginning of the recording. Fluorescent intensity is shown in color. White arrows in the first image indicate the direction of the puff application of Ach. Scale bar: 100 μm. (A2 and B2) Images of the myocytes analyzed in Fig 3. B1, B2, B3, C1, C2 and C3 correspond to the fibers from B1 to C3, respectively. Scale bar: 100 μm. (TIFF) [file pbio.3003137.s007.tiff]

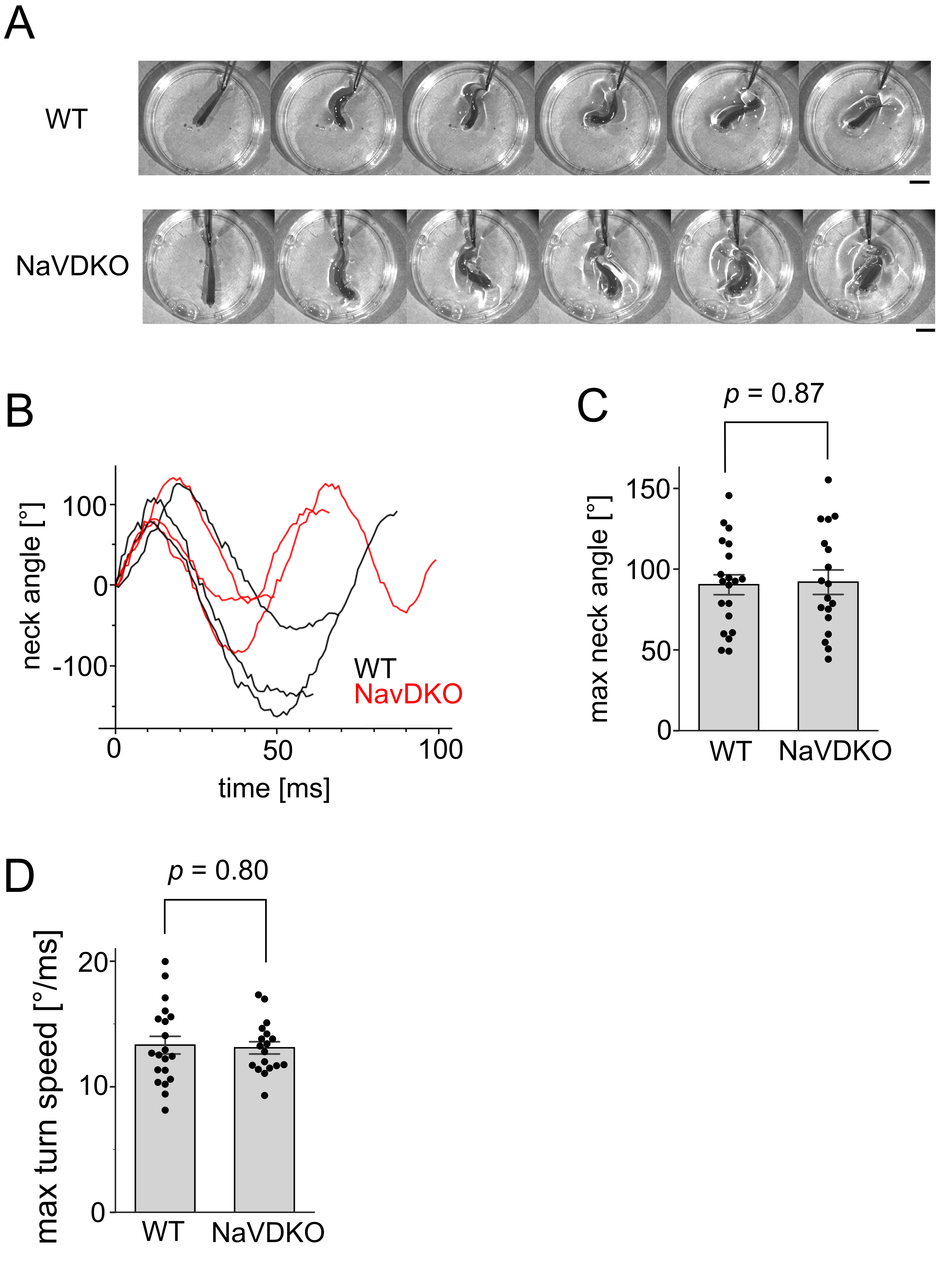

Supplement: S8 Fig — (A) Representative escape responses of adult WT (upper panels) and NaVDKO (lower panel) fish. Images were captured every 10 ms. Scale bar: 1 cm. (B) Representative plots of the “neck angle” during the escape behavior of WT (black) and NaVDKO (red) fish. (C) Maximum neck angles of WT and NaVDKO fish. The maximum angle within 40 ms of the start of the response is plotted (n = 20 for WT and n = 18 for NaVDKO). (D) Maximum turn speed in WT and NaVDKO fish (n = 20 for WT and n = 18 for NaVDKO). The numerical data presented in this figure can be found in S1 Data. (TIFF) [file pbio.3003137.s008.tiff]

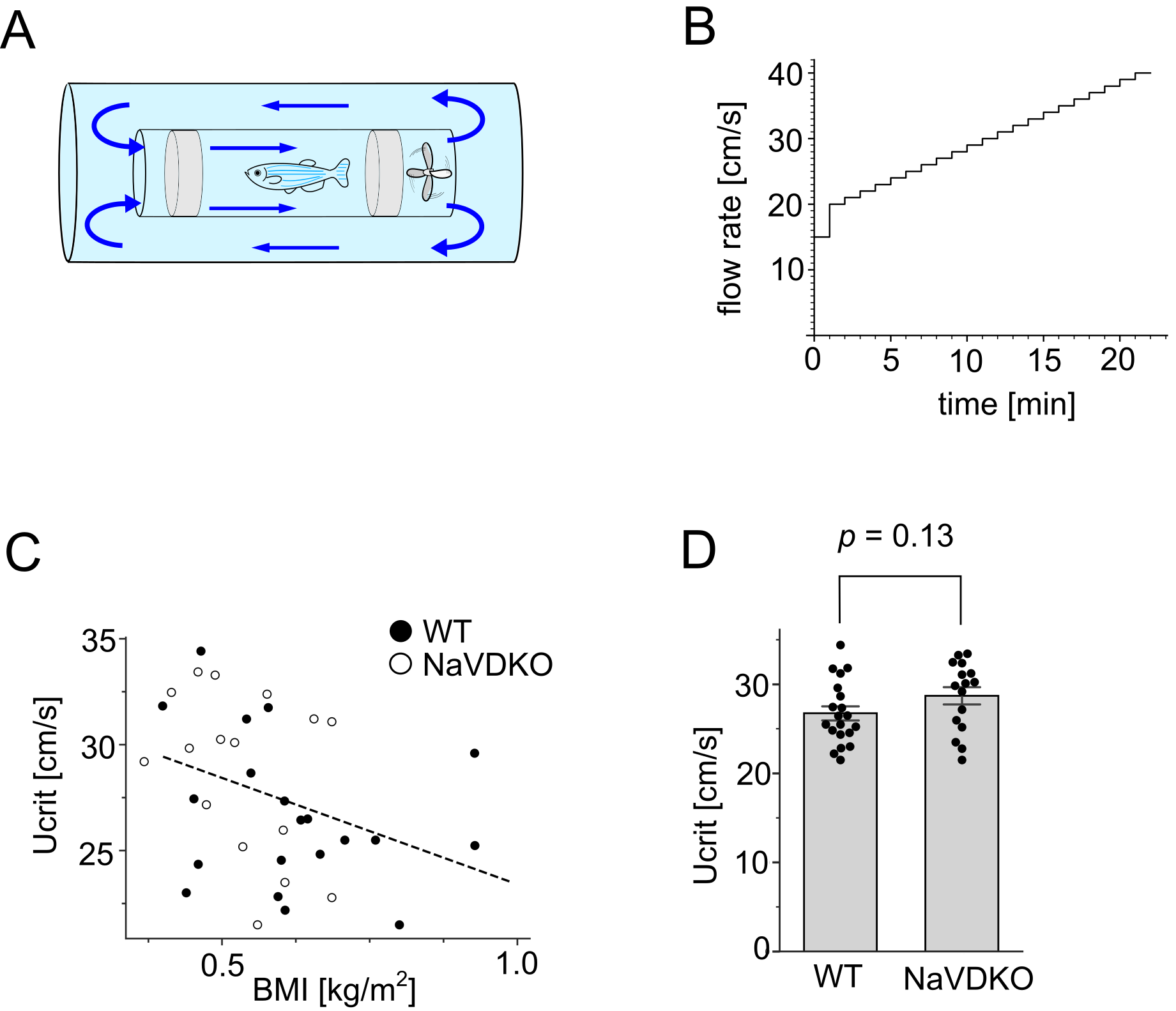

Supplement: S9 Fig — (A) Schematic of swimming treadmill. (B) Swimming treadmill protocol. The flow rate was initially set at 15 cm/s for 1 min, followed by increments of 1 cm/s every min, starting at 20 cm/s. (C) Critical swimming speed (Ucrit) of individual fish plotted against the BMI. Open and closed circles represent WT and NaVDKO fish, respectively (n = 20 for WT and n = 16 for NaVDKO). Dotted line represents regression line. Pearson’s correlation coefficient was −0.35. (D) Comparison of Ucrit values between WT and NaVDKO fish (n = 20 for WT and n = 16 for NaVDKO). The numerical data presented in this figure can be found in S1 Data. (TIFF) [file pbio.3003137.s009.tiff]

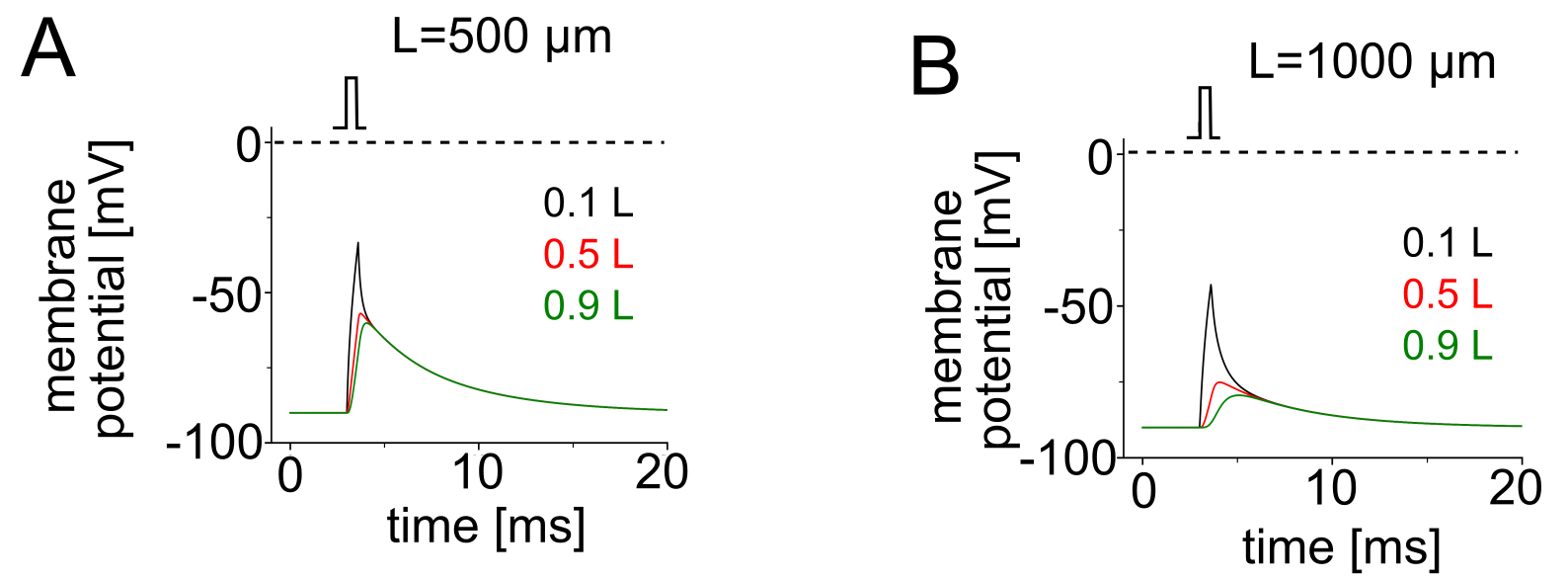

Supplement: S10 Fig — (A and B) Membrane potentials with fiber lengths (L) of 500 (A) and 1,000 μm (B). Potentials were calculated based on the geometry shown in Fig 4A. The numerical data presented in this figure can be found in S1 Data. (TIFF) [file pbio.3003137.s010.tiff]

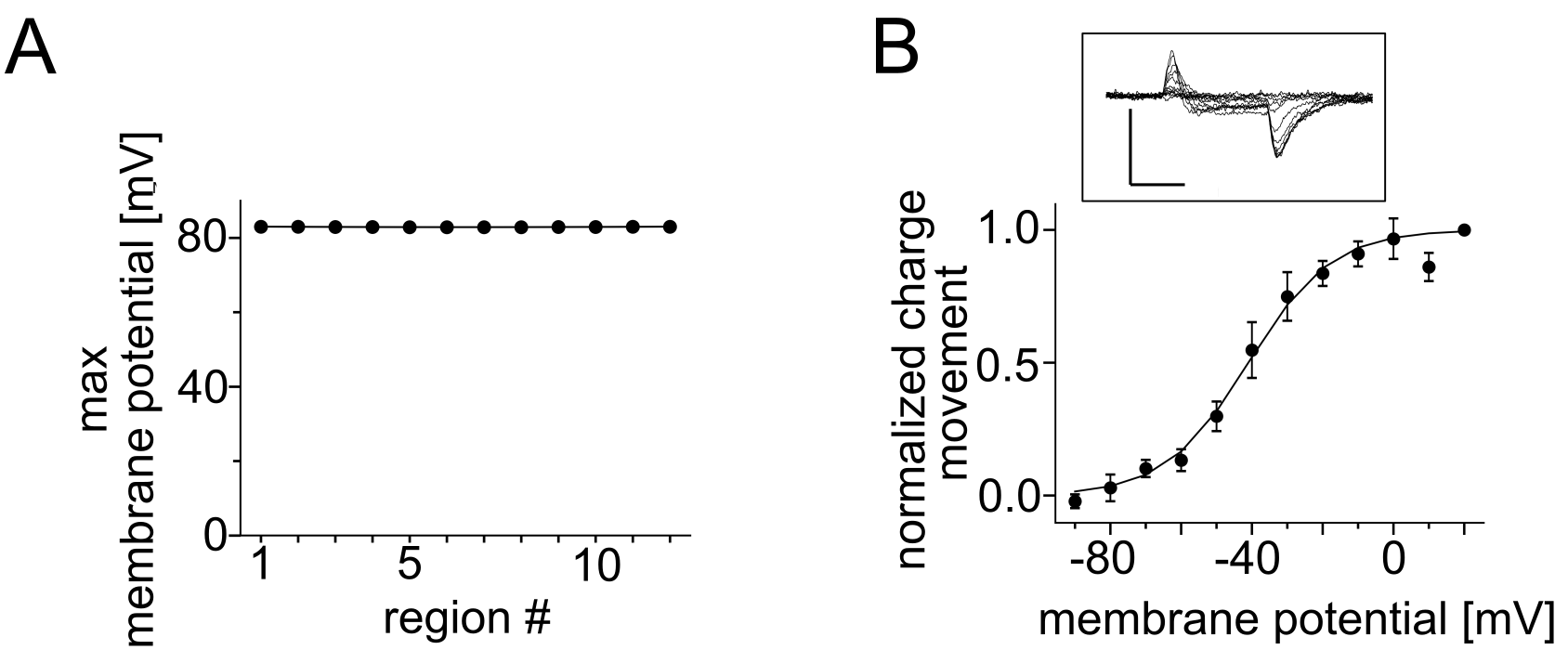

Supplement: S11 Fig — (A) Maximum potentials at the center of each region in the model shown in Fig 4F. The maximum amplitudes were well above 50 mV in all regions. (B) “Gating” current of DHPR in WT fish. The charge movement of the OFF current plotted against the membrane potential. Data shown as mean ± SEM (n = 5) and fitted with the Boltzmann equation. The inset shows representative traces of the “gating” current. Traces evoked by voltage steps ranging from −90 mV to 20 mV in 10 mV increments. The vertical and horizontal scale bars indicate 0.5 nA and 5 ms, respectively. The numerical data presented in this figure can be found in S1 Data. (TIFF) [file pbio.3003137.s011.tiff]

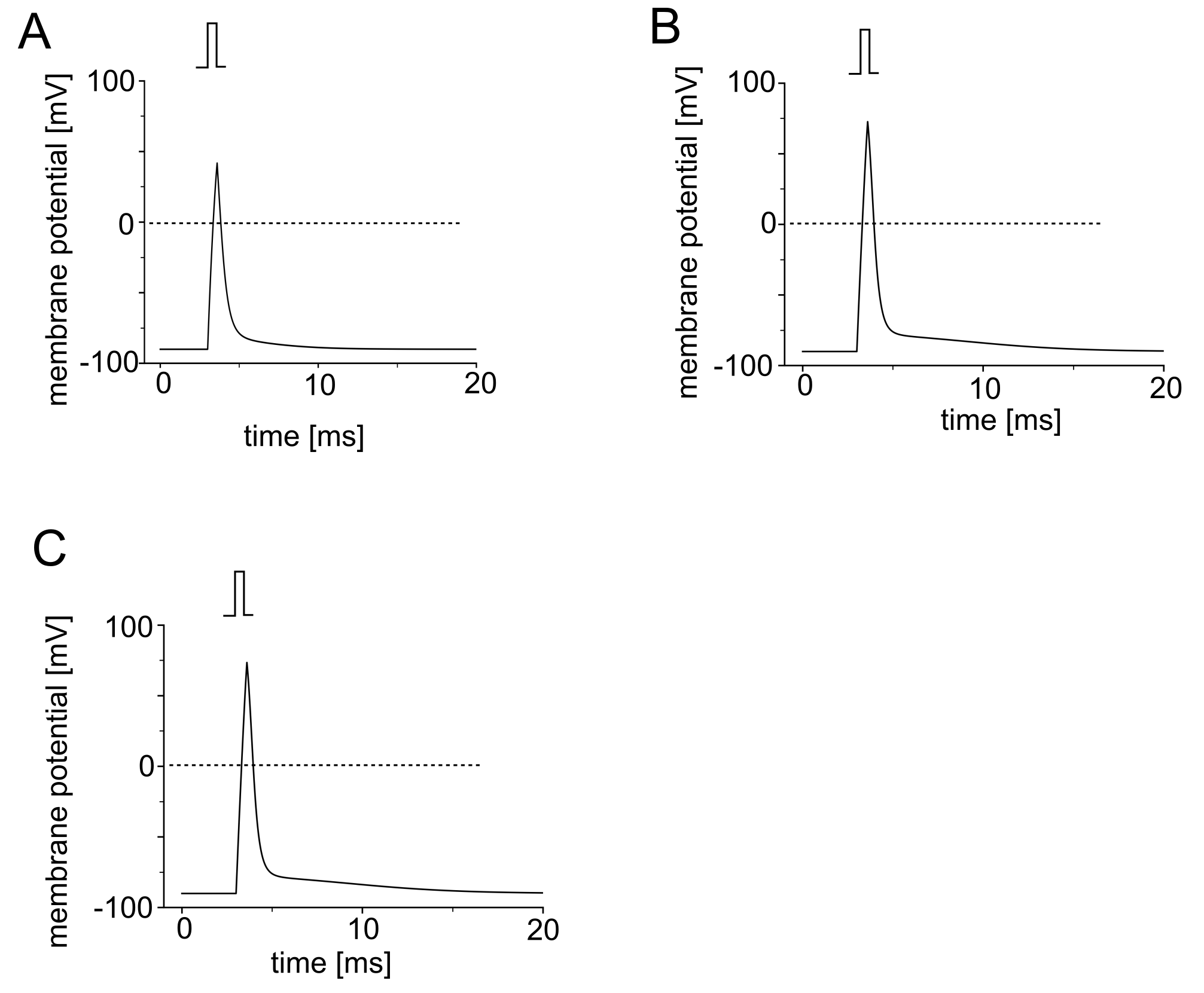

Supplement: S12 Fig — (A, B, and C) Membrane potential at the center (0.5 L) of region #6 (Fig 4F) calculated using the parameters obtained from the squid giant axon (A), lobster leg axon (B), and goldfish Mauthner axon (C). The resistivity values are shown in S7 Table. The mean values were used for the simulation. The panels above the plots indicate the timing of the synaptic input. The numerical data presented in this figure can be found in S1 Data. (TIFF) [file pbio.3003137.s012.tiff]

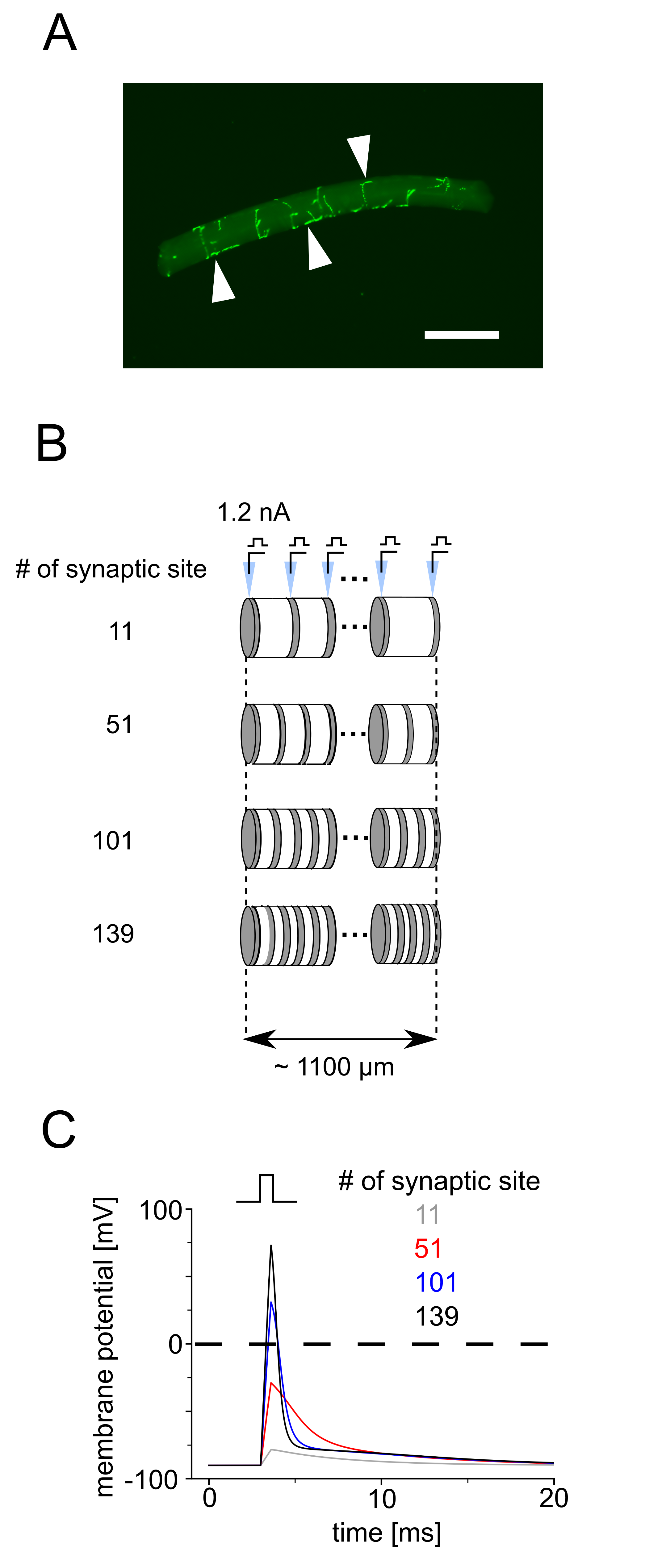

Supplement: S13 Fig — (A) Representative images of adult WT myofibers. Scale bar: 200 μm. Arrowheads indicate the linear ACh release sites. (B) Simulation of adult fibers. Total length of the fibers was approximately 1,100 µm, with ACh release site numbers ranging from 11 to 139. The current amplitude at the individual ACh release sites was set to 1.2 nA. (C) Membrane potential at the center of the fiber in each model. The square pulses above the plots indicate the timing of the synaptic input. The numerical data presented in this figure can be found in S1 Data. (TIFF) [file pbio.3003137.s013.tiff]
