# Supplementary material for: Normal locomotion in zebrafish lacking the sodium channel NaV1.4 suggests that the need for muscle action potentials is not universal
Source: PLoS Biol. 2025 Apr 24;23(4):e3003137. doi: 10.1371/journal.pbio.3003137 (PMC12021243; doi:10.1371/journal.pbio.3003137)
Supplement: S1 Table — Annotations and nomenclatures were derived from the Ensemble release113. (DOCX) [file pbio.3003137.s014.docx]

S1 Table. Voltage-gated sodium channels in human and zebrafish

| Protein | Human gene | Zebrafish gene |
| --- | --- | --- |
| **α subunits** |  |  |
| NaV1.1 | *SCN1A* | *scn1aa, scn1ab* |
| NaV1.2 | *SCN2A* |  |
| NaV1.3 | *SCN3A* |  |
| NaV1.4 | *SCN4A* | *scn4aa, scn4ab* |
| NaV1.5 | *SCN5A* | *scn5lab* |
| NaV1.6 | *SCN8A* | *scn8aa, scn8ab* |
| NaV1.7 | *SCN9A* |  |
| NaV1.8 | *SCN10A* |  |
| NaV1.9 | *SCN11A* |  |
|  |  | *scn12aa* |
| β-subunits |  |  |
| β1, β1B | *SCN1B* | *scn1ba, scn1bb* |
| β2 | *SCN2B* | *scn2b* |
| β3 | *SCN3B* | *scn3b* |
| β4 | *SCN4B* | *scn4ba, scn4bb* |

Annotations and nomenclatures were derived from the Ensemble release113.
