# Supplementary material for: Normal locomotion in zebrafish lacking the sodium channel NaV1.4 suggests that the need for muscle action potentials is not universal
Source: PLoS Biol. 2025 Apr 24;23(4):e3003137. doi: 10.1371/journal.pbio.3003137 (PMC12021243; doi:10.1371/journal.pbio.3003137)
Supplement: S5 Table — (DOCX) [file pbio.3003137.s018.docx]

S5 Table. α1-subunits of voltage-gated calcium channels in human and zebrafish

| Protein | Human gene | Zebrafish gene |
| --- | --- | --- |
| CaV1.1 | *CACNA1S* | *cacna1sa, cacna1sb* |
| CaV1.2 | *CACNA1C* | *cacna1c* |
| CaV1.3 | *CACNA1D* | *cacna1da,cacna1db* |
| CaV1.4 | *CACNA1F* | *cacna1fa, cacna1fb* |
| CaV2.1 | *CACNA1A* | *cacna1aa, cacna1ab* |
| CaV2.2 | *CACNA1B* | *cacna1ba, cacna1bb* |
| CaV2.3 | *CNCNA1E* | *cacna1ea,cacba1eb* |
| CaV3.1 | *CACNA1G* | *cacna1g* |
| CaV3.2 | *CACNA1H* | *cacna1ha, cacna1hb* |
| CaV3.3 | *CACNA1I* | *cacna1ia* |

Annotations and nomenclatures were derived from the Ensemble release113.
