# Supplementary material for: Normal locomotion in zebrafish lacking the sodium channel NaV1.4 suggests that the need for muscle action potentials is not universal
Source: PLoS Biol. 2025 Apr 24;23(4):e3003137. doi: 10.1371/journal.pbio.3003137 (PMC12021243; doi:10.1371/journal.pbio.3003137)
Supplement: S7 Table — Average values are shown in brackets. Note that the specific leakage conductance is inverse of the resistivity of the membrane. (DOCX) [file pbio.3003137.s020.docx]

S7 Table. Diameter and resistivitis of neurons and muscle fibers.

|  | Squid giant axon | *H.vulgaris* (lobster) leg axon | *R. temporaria* (frog) extensor longus dig. IV | Gold fish Mauthner axon |
| --- | --- | --- | --- | --- |
| Diameter [µm] | 360-540 | 62-87 (75) | (45) | 54 |
| Resistivity of the membrane  [Ωcm^2^] | 500-110 | 564-4590  (2290) | 1500-9500  (4300) | 2200 |
| Resistivity of fiber interior  [Ωcm] | 22-34 | 43.2-83.6  (60.5) | 206-355  (255) | 120 |

Average values are shown in brackets. Note that the specific leakage conductance is inverse of the resistivity of the membrane.
