# Supplementary material for: Normal locomotion in zebrafish lacking the sodium channel NaV1.4 suggests that the need for muscle action potentials is not universal
Source: PLoS Biol. 2025 Apr 24;23(4):e3003137. doi: 10.1371/journal.pbio.3003137 (PMC12021243; doi:10.1371/journal.pbio.3003137)
Supplement: Attachment — Submitted filename: Response to Reviewers.pdf [file pbio.3003137.s032.pdf]

> Reviewer #1:

>

> Akiyama et al. present a description of the unperturbed locomotion of zebrafish harbouring mutations in scn4aa and scn4ab, which encode Nav1.4s, the voltage-gated sodium channels involved in generating action potentials in skeletal muscle in excitation-contraction coupling. Using a combination of imaging, electrophysiology and in silico modelling, the authors present evidence that action potentials facilitated by Nav are dispensable in its role coupling excitation by ACh to eventual contraction in zebrafish muscle fibres. These findings are particularly intriguing, as they stand against established dogma of the role of these proteins in generating action potentials in mammalian muscle. Thus, the subject matter, scope and findings of the presented short manuscript, despite in essence being mainly composed of negative results, are appropriate for publication in PLOS Biology's 'short reports'. However, in its current state, this manuscript is not yet of a standard to be

> published.

>

> To bring this manuscript up to a standard suitable for inclusion in PLOS Biology as a 'short report', a number of issues should be addressed. Generally, these can be easily addressed by reworking the figures and a more thorough proofread.

>

> For a less expert audience I think it would be good to explain a bit more about the Voltage-gated sodium channel gene family, its expression and roles, and why Nav1.4 is so critical for AP generation in skeletal muscle biology. There is a bit too much implied knowledge in the introduction and more context is needed.

Thank you for the suggestion. We have included the suggested description of the Nav subfamily in the Introduction, with an emphasis on beta subunits, which Reviewer #2 pointed out as a major concern. Additionally, the new sentences highlight the significance of AP and NaV1.4 in skeletal muscles.

> Leading on from this point perhaps the authors can better explain for instance why can't another Voltage-gated sodium channel be acting redundantly in muscle in zebrafish to scn4a/b? Are these the only relevant Voltage-gated sodium channels expressed at the time and place. Are other channels up regulated in the mutants? Does TTX block all

channels equally? I know the in vitro recordings argue against this but I thought this needed a bit more context to be accessible.

Thank you for pointing this out. Next-generation sequencing of trunk muscles from adult WT and NaVDKO fish was performed to examine whether other NaVs were upregulated. The data were added as Fig. S4 and Tables S2–S4 and S6, and the corresponding discussion is added in the Results section. In combination with the physiological data, we can now say with confidence that the knockout of NaV1.4 is not compensated for by the upregulation of other NaV or CaV genes.

> In line 319, it is stated that 'zebrafish myofibres lack Ca2+ conductance (Fig. 1A)'; however, this is not shown in fig. 1a.

We confirmed previous reports (ref 17, 28) that CaV currents could not be recorded in myofibers (Fig. 1C and 1D), which was also observed in NaVDKOs.

> In line 228, 'L' should be defined in the text.

The definition of L as the length of the model fiber has been added in line 475-476, page 15 in the revised manuscript.

>Please clarify the statement in line 358, as in line 354, 'quantal content' was defined as 'all synaptic sites', but the statement in line 358 would be redundant given the previous statement of equivalence of the number of synaptic sites and quantal content.

We re-wrote the sentences to clarify the logic in line 491- 498, page 16.

> In the methods section, some additional information is required. In lines 92-93, it is stated where zebrafish were kept, but not how. Were fish maintained under standard conditions?

The description on the maintenance of the fish is added in line 108 – 111, page 5.

> In line 119, more detail is needed about the high-speed camera used. At minimum, the model used should be stated. Objective information where known would also be helpful. In line 120, it is stated that measurements were made by ImageJ. The tools or plugins

used should be stated, along with their applicable version numbers. The 'curve' mentioned in line 121 should be defined. 'NMDA' in line 135 should be defined. Please state the version of the 'PatchMaster' software used (line 173) and which programmes were run on python (line 174).

The suggested change has been made.

> A number of grammatical and proofreading errors are present and impede readability of an otherwise interesting manuscript.

We apologize for the errors in the original manuscript. We have carefully checked and eliminated these errors in the revised version.

> Gene nomenclature is not properly or consistently used. Genes should be emphasised (scn4aa and scn4ab, lines 78, 98 and so forth). If referring to human genes, they should be additionally capitalised, see line 57, which should read as SCN4A, as a human gene is being referred to. A good zebrafish-focused reference is the ZFIN zebrafish nomenclature guide.

As suggested, we have emphasized *scn4aa* and *scn4ab* throughout the manuscript, following the guidelines of the ZFIN (Zebrafish Information Network.

> When referencing figures, numbers should go after the term 'fig.', not the other way around (lines 254, 255, 256 258 &c.).

Referencing style such as “S1 Fig” seems common in PLOS Biol. However, we agree that they sound strange. We will follow instructions from the editorial office.

> In line 69, it is stated that two major kinds of muscle 'cells' are found in vertebrate skeletal muscle, this should instead read 'fibres'.

The correction has been made to replace “cells” with “fibres,” as suggested in line 88, page 4 in the revised manuscript.

> The figures as they currently stand are difficult to interpret and would benefit greatly from the addition of legends. In figure 1a, the three current traces are given without context. Simply adding 'WT', 'WT + TTX' and 'DavDKO' to their corresponding traces would greatly improve comprehensibility. Similarly, given the ample space, figure 2a

could be better annotated to indicate that panels are showing a process over time and how much time is passing. This is also applicable for the panels in fig. 3. In figure 2b, the schematic in the inset is made greatly more challenging to read by having schematic elements in black, making them hard to distinguish from the zebrafish. Use colour and annotations. Also the y-axis label should read 'neck angle [°] here.

Thank you for the helpful suggestions. The figures have been modified with appropriate annotations and the y-axis label has been updated to read 'neck angle [°]' as suggested.

> In figure 3, there should be some indication on the figure that the fluorescence is that of GCaMP.

In Figure 3A, we have added an indication that the fluorescence corresponds to GCaMP.

> Additionally, it would be much easier to interpret changes in GCaMP intensity by using a multi-colour LUT, such as the 'fire' LUT in ImageJ.

Thank you for the excellent suggestion. We now present GCaMP imaging using the ‘fire’ LUT in ImageJ.

> Should the legend in figure 3d read 'C1', 'C2' &c. instead of 'G1', 'G2' and so forth?

The errors have been corrected in the figure legend, changing 'G1', 'G2', etc., to 'C1', 'C2', etc.

> Instead of showing the control condition (without TTX) in the supplementary figures, it should be in figure 3. Panels A & B of fig. 3 should be in turn moved to the supplementary figures.

The figures have been updated. The control condition (without TTX) has been moved to Figure 3, and panels A and B of Figure 3 have been moved to the supplementary figures as requested.

> Like with other figures, figure 4 needs more annotation, namely an indication of which condition is shown (B, C & D). The panels in fig. 4c should indicate what is being stained and are difficult to see. Please include panels with greater magnification to better see the staining along these fibres.

> We have added annotations and included larger magnification

panels in Fig. 4 for better visibility of the staining along the fibres. For Fig. 4B, three data points (0.1, 0.5, and 0.9 L) were overlapped. We have mentioned this in the legend.

>

>

>

> Reviewer #2:

>

> This short report investigates the impact of knocking out Nav1.4aa and 1.4ab on fast-twitch muscle contraction and the resultant impacts on both larval and adult swimming. The main conclusion of the paper is that the role of sodium channels in muscle contraction is not conserved. There are some issues with this very densely written manuscript and uninformative figures. The main conclusion of the authors is that sodium channels are not required for muscle contraction. The data shown indicate that double mutant embryos and adults swim normally and exhibit calcium currents. Modeling suggests that EPPs could be sufficient for contraction. However, this reviewer is concerned about a couple of things. One, there is so little explanation in the methods as to not allow the reader to assess the results (for example, how were fish ID'd - see below). For example, only some of the cell numbers resulted in EPPs being sufficient for contraction - why is this?

>

> Two, using Daniocell one can view the relative expression levels of sodium channels during development. Scn4aa is barely expressed in only a few cells, and scn4ab is also expressed at a very low level but in slightly more cells. Thus, in general, the idea that double knockouts of these genes are not only not lethal but not really impactful on swimming is not unpredicted in this case. In contrast, scn4ba is expressed at MUCH higher levels in MANY more cells. Scn4bb is also expressed at higher levels in more cells than the alpha variants. It is critically important, if the authors want to conclude that sodium channels are dispensable for contraction, then it is fundamentally important to address these much more highly expressed variants.

We thank the reviewer for pointing this out. As Reviewer #1 pointed out, we should have provided more background information on voltage-

gated sodium channels in general.

Scn4ba **and** ***Scn4bb*** **encode the beta subunits of NaVs but not the alpha subunits. The beta subunits contain a single non-voltage-sensitive transmembrane domain without ionic pores to conduct sodium ions and modify the gating of the alpha subunits. Although the beta subunits are more widely and abundantly expressed than the alpha subunits, their products cannot function as ion channels in the absence of alpha subunits.** ***Scn4aa*** **and** ***scn4ab*** **are the only alpha subunit genes expressed in significant amounts in skeletal muscles. In the revised version, we have discussed these points in the Introduction and Results sections.**

> The figures are not friendly to a casual reader. Everything should be labeled so that one can understand the figure without reading the text.

We agree that the annotation was insufficient, as Reviewer #1 also pointed out. We have increased annotations as much as we can.

>In supp figure 1 please highlight the impact of the mutations - presumably at some point there is a stop codon, where does this occur? What is known about these different domains of these channels? Is this guaranteed to be a null?

The reading frames and overall structure of the NaV alpha subunit are shown in Fig. S1. Stop codon locations are shown in the diagram.

> One important detail is how double mutants were ID'd. There is one mention that carriers were identified by sequencing but how were the embryos ID'd? If cells were isolated from 3-8 day old fish, how were the fish/cells genotyped? Was it all done by sequecing after the fact? Was the ZEG used? Were specific primers used?

We apologize for this lack of information. These procedures are now described in detail in the Methods section.

>

>

> Reviewer #3:

>

> The present study challenges the long-standing belief that action potentials are essential for muscle contraction by examining zebrafish with mutations in the scn4aa and scn4ab genes, which encode the Nav1.4 sodium channels. Using CRISPR-Cas9, the

researchers created zebrafish lacking these channels (NavDKO). Surprisingly, both embryos and adult mutant fish displayed normal escape responses and locomotion, comparable to wild-type fish.

\>

> Through calcium imaging, they discovered that acetylcholine could still excite myofibers, even when sodium channels were blocked with tetrodotoxin. This indicates that Nav channels are not required for muscle contraction in zebrafish. Mathematical modeling further suggested that the small size of the muscle fibers and the widespread distribution of neuromuscular synapses enable sufficient depolarization to trigger muscle contraction without the need for Nav1.4.

\>

> Thus, the study concludes that Nav1.4 channels are not essential for muscle contraction in zebrafish, and the physiological role of these channels in muscle function is not uniform across vertebrate species. Overall this is a novel and provocative study.

\>

> In my opinion few main points need to be improved.

\>

> 1) The calcium imaging data needs a more rigorous quantification.

Thank you for your comments. Following the comments of Reviewer #1, we have indicated the fluorescence intensity using a color code. For quantitative presentation, time-dependent changes in fluorescence intensity were plotted without normalization in Figs. 3 and S6.

> 2) The development and maturation of NMJ in mutants and wild type should be analyzed at various embryonic and larval stages to check if compensatory mechanisms are in place in the mutant. This can be done with classical immunohistochemistry staining (i.e. SV2 and alphaBTX staining). See also https://doi.org/10.1093/bioinformatics/btad720 or doi: 10.3389/fnmol.2022.956582

Thank you for this helpful suggestion. We performed double staining for SV2 and alpha BTX in 28 hpf, 3 dpf, and 5 dpf embryos. The anatomy of the NMJs was not distinguishable between WT and NaVDKO embryos. These data are included in the revised manuscript as Fig. S5.

> 3) Finally as compensatory mechanisms may hide the phenotype at later stages the authors should check the earliest locomotor behavior of embryonic zebrafish, the spontaneous coiling that embryos perform inside the chorion starting at 18hpf and

quantify this behavior.

Thank you for pointing this out. We recorded the coiling activity in approximately 30 hpf embryos. These data have been included as Fig. S2 in the revised manuscript.
